# Management of subepithelial esophageal tumors

**DOI:** 10.1515/iss-2023-0011

**Published:** 2024-08-20

**Authors:** Marcel A. Schneider, Diana Vetter, Christian A. Gutschow

**Affiliations:** Department of Visceral and Transplant Surgery, University Hospital Zürich, Zurich, Switzerland

**Keywords:** esophagus, tumor, benign, surgery, GIST, leiomyoma

## Abstract

Subepithelial esophageal tumors (SET) are normally benign intramural esophageal lesions of mesenchymal origin. Although rare, the incidence of SET has increased in recent decades due to the more widespread use of endoscopy and diagnostic imaging. The current review aims to provide an overview of the histopathologic spectrum and the most frequent entities including leiomyoma and gastrointestinal stromal tumor (GIST), diagnostic workup, and multidisciplinary treatment options. Staging for SET should include endoscopy, endoscopic ultrasonography (EUS), and tissue sampling. Current consensus guidelines recommend that SET suggestive of gastrointestinal stromal tumor (GIST) larger than 20 mm or lesions with high-risk stigmata should undergo tissue sampling. Most SET have an excellent long-term outcome, but malignancy may be present in certain subtypes. Asymptomatic SET without high-risk stigmata discovered incidentally usually do not require specific treatment. However, depending on the size and location of the lesion symptoms may occur. Therapeutic interventions range from endoscopic interventional resections to major surgical procedures. Enucleation via minimally invasive or robotic-assisted access remains the standard of care for most SET sub-entities.

## Introduction

Subepithelial esophageal tumors (SET) are usually benign intramural esophageal lesions of mesenchymal origin covered by normal-looking mucosa and protruding into the lumen. SET are generally rare, occurring in postmortem series with an incidence of 0.5–1 % [1], [2], [3]. SET are much less common than malignant epithelial esophageal tumors [4], [5], [6] and account for less than 5 % of all surgically treated esophageal neoplasms [3], [6], [7], [8]. Of note, the incidence has increased in recent decades, most likely related to a more widespread use of endoscopy and diagnostic imaging [9], 10], while also showing some geographical variation [11].

SET is an umbrella term for several subtypes, and a solid knowledge of the histopathologic spectrum, diagnostic workup, and treatment options is essential. Therefore, treatment planning involving a multidisciplinary tumor board is generally recommended. Small asymptomatic SET usually requires only close surveillance, whereas therapy may be indicated based on symptoms, tumor size and growth, or an unclear diagnosis. Most SET have an excellent long-term outcome, but malignancy may be present in GIST and, less commonly, in Schwannoma, granular cell tumor, or even leiomyoma. Therapeutic interventions range from endoscopic interventional resections to major surgical procedures such as esophagectomy and multimodal approaches. However, enucleation via a minimally invasive or robotic-assisted access remains the standard of care for most SET sub-entities.

Given the increasing incidence and the growing demand for a refined management of SET, this review aims to provide a topical update with a focus on treatment strategies. Esophageal neuroendocrine neoplasms (NEN) and esophageal malformations such as cysts and duplications may also manifest clinically as SET. However, due to the high malignant potential of esophageal NEN and the non-neoplastic nature of esophageal malformations, these entities fall outside the scope of this narrative review.

## Pre-therapeutic diagnostic work-up

Staging for SET should include endoscopy, endoscopic ultrasonography (EUS) and tissue sampling. Endoscopy is essential for macroscopic evaluation of the lesion, accurate pretherapeutic determination of the anatomic location, and histopathologic diagnosis. Endoscopically, SET typically appear as roundish luminal bulging lesions of variable size and texture with or without a central umbilication; the consistency usually is soft and may be roughly assessed with biopsy forceps [12], 13]. Magnifying endoscopy or chromoendoscopy is of limited value because of the normal overlying mucosa, but some lesions may show through in whitish-grey or reddish-brown color. Larger lesions, rapid growth, or ulceration are indicators of possible malignant transformation [14].

EUS is the diagnostic tool of choice to characterize important features such as size and shape, exact location, originating layer and echogenicity [12]. The most recent ESGE guidelines recommend EUS-guided fine-needle biopsy for all SET suggestive of GIST, greater than 2 cm in diameter, and with high-risk stigmata. In addition, EUS allows reliable differentiation of extrinsic compression [12], 15] and can determine the intramural layer of origin, the size and the margins of the lesion with high accuracy [15], 16]. However, except for lipomas and hemangiomas, the classic EUS features such as homogeneity, spots, halo, and echogenicity are not specific for most SET subtypes [17], [18], [19].

In our institutional routine, cross-sectional radiologic imaging with thoraco-abdominal 3-phase CT-scan is also performed [20]. In selected cases – particularly GIST (which are often FDG positive), suspected malignancy, or large tumors – PET-scan or MRI can provide valuable additional information for oncological staging and therapeutic planning [21], 22].

### Histopathological assessment

Current consensus guidelines recommend that SET suggestive of GIST larger than 20 mm or lesions with high-risk stigmata should undergo tissue sampling to assess risk of progression before any therapeutic decision is made [12]. Likewise, in symptomatic tumors (except for SET with acute hemorrhage), a definitive diagnosis should be made before treatment, regardless of size. The need for a histopathological diagnosis is debated for small asymptomatic SET <20 mm without high-risk features [12], 23], which can also be followed up endoscopically according to the National Comprehensive Cancer Network guidelines [24]. However, because most national and international guidelines [25], [26], [27] recommend surgical resection of small esophageal GIST <20 mm, it is our institutional policy to strive for a definite diagnosis even in these situations [14]. As recommended in the current European Society for Gastrointestinal Endoscopy (ESGE) guideline, tissue sampling for SET ≥20 mm can be performed using either EUS-guided fine-needle biopsy or mucosal incision-assisted biopsy (MIAB), whereas tumors <20 mm should be exclusively biopsied using MIAB [12].

## Spectrum of SET and indication for therapy

### Leiomyoma

Leiomyomas are usually diagnosed between the 3rd and 5th decades of life [3], 7], 28], 29]. They are the most common benign neoplasms of the esophagus, accounting for approximately two-thirds of all SET [1], 3], 4], 30], 31]. Leiomyoma usually grow in the middle and distal esophageal thirds because they develop from smooth muscle cells and are less common in the upper third, where the muscle layer is primarily striated [6]. Most leiomyomas develop from the muscularis propria of the esophagus and present as a subepithelial mass; however, cases arising from the muscularis mucosae may manifest luminally with a polyp-like growth pattern [32].

Esophageal leiomyomata are often small, reflecting their slow growth, with approximately 50 % <5 cm and 93 % <15 cm^7^. Symptomatic tumors have usually reached a size of at least 5 cm^3^. Endoscopically, leiomyomas present as whitish-gray to brown–yellow, rubber-like, encapsulated masses with a smooth surface [6]. An irregular, often dendritic, horseshoe- or dumbbell-shaped growth pattern is typical, and even smaller lesions may occasionally present with clamp-like narrowing of the lumen.

Histologically, spindle-shaped cells arranged in fascicles or whorls are typically found. The cytoplasm is eosinophilic with surrounding hypovascular connective tissue [33]. There are few or no mitoses, with minimal nuclear atypia and general hypocellularity. Definitive diagnosis of leiomyoma is based on positivity of the immunohistochemical markers desmin and smooth muscle antigen (SMA) [33]. Malignant transformation to leiomyosarcoma is extremely rare, with fewer than 200 cases reported [6], 31], 32], 34]. Esophageal leiomyosarcomas tend to grow intraluminally rather than intramurally and have a 5-year survival rate of 20 % [32].

Treatment is indicated in all symptomatic cases. Minimally invasive surgical enucleation is the treatment of choice [35], because it involves fewer pulmonary complications, pain, and hospitalization compared with an open procedure [36]. However, extended resection techniques may be required for giant leiomyomas [37]. In asymptomatic cases, endoscopic surveillance at 6–12-month intervals is recommended.

### Gastrointestinal stroma tumor (GIST)

Less than 1 % of GIST is found in the esophagus – compared with 50–60 % in the stomach and 30–35 % in the small intestine [38]. GIST arises from the interstitial cells of Cajal, also known as the gastrointestinal motility pacemaker [39]. Most esophageal GIST (eGIST) are diagnosed between the fifth and seventh decade [34] are variable in size and develop primarily in the distal third. Clinically, eGIST may present with gastrointestinal bleeding, but also with dysphagia and weight loss.

**Figure 1:**
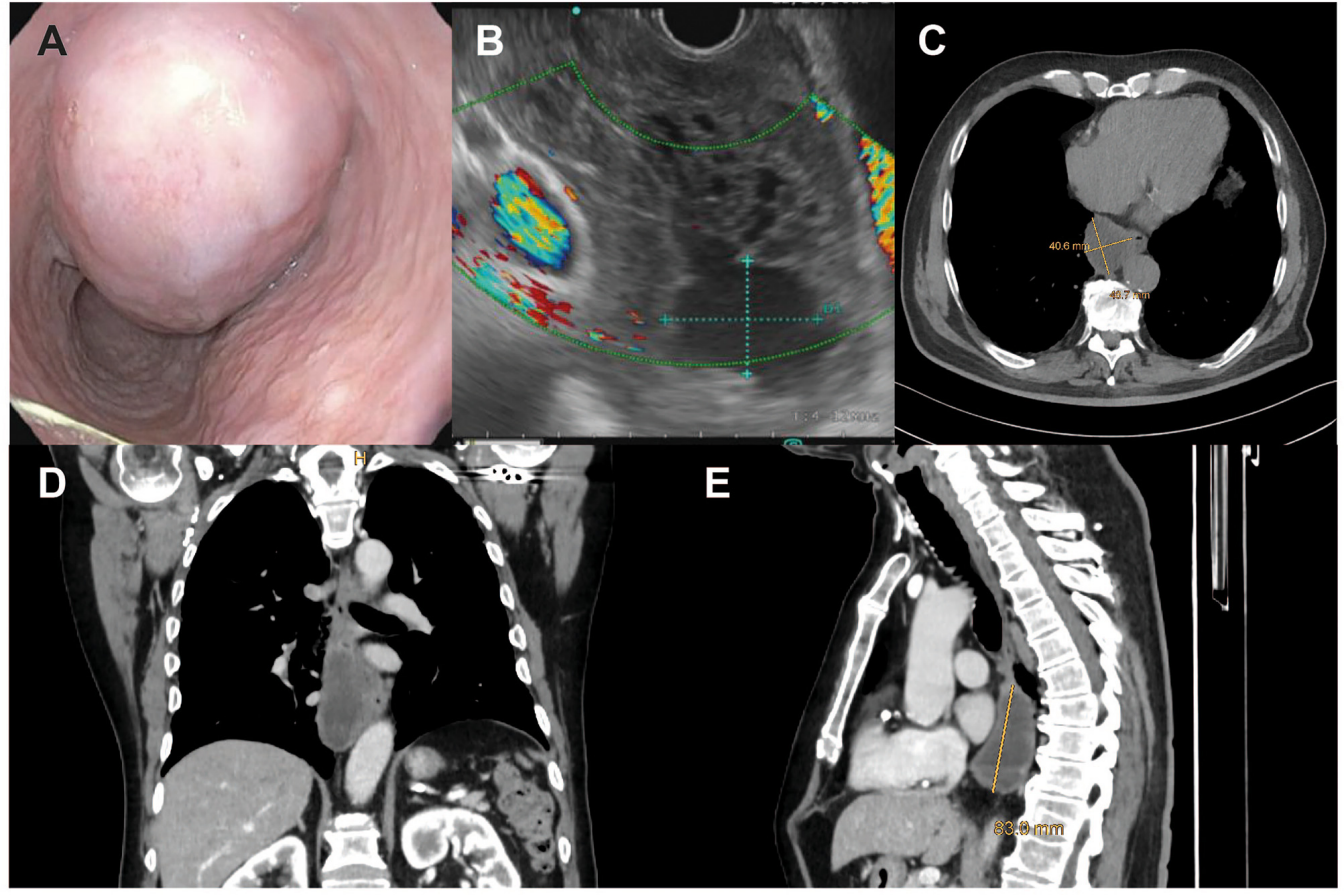
Case of a male patient with a large (9 × 5 cm) esophageal GIST. Endoscopic (A) and endosonographic (B) view, as well as transverse (C), coronal (D) and sagittal (E) computed tomography planes of the tumor.

The gross appearance of the tumor is usually whitish gray, soft, encapsulated, and fish-flesh-like (Figure 1). Histologically, GIST has an overall basophilic appearance with high cellularity and mild-to-no nuclear pleomorphism on hematoxylin-eosin staining. Total of 70–80 % are composed of spindle cells, while 20–30 % are predominantly epithelioid. On immunohistochemistry, the most common markers are c-kit protein (CD117), DOG-1, and CD34, with CD117 being the most specific [33]. Mitotic activity plays a crucial role in predicting malignant potential. The National Institute of Health (NIH) has developed a classification system for GIST in general with four risk categories based on mitotic activity and size, specifically very low risk, low risk, intermediate risk, and high risk [40]. In 2016, Miettinen from the Armed Forces Institute of Pathology (termed AFIP classification), introduced a classification consisting of six groups further including the anatomic site of the primary tumor as an additional variable [41]. The most recent classification by Joensuu et al., also includes the additional risk of tumor rupture and proposes a continuous risk scale [42]. The used non-linear model accurately predicts the risk of recurrence and can be nicely visualized using prognostic contour maps. All of these risk prediction models distinguish well between high-risk and low-risk patients with resected, non-metastasized GIST [43]. Additionally, it has recently been shown that certain KIT deletion mutations in exon 11 are associated with recurrence and metastasis in eGIST [44].

Because of their inherent malignant potential, eGIST require close clinical monitoring or, if medium- or high-risk, surgical resection. R0 resection remains the aim to strive for during surgery, while lymphadenectomy, as for GIST at other localizations, is usually not required [45]. Imatinib [46] or other tyrosine kinase-inhibitors such as sunitinib, regorafenib [47] (in case of wildtype KIT/PDGFR-A) or avapritinib (in case of wildtype PDGFRA-D842V mutation) [48] can be used as systemic neoadjuvant [49] or adjuvant [50] treatment. According to the latest ESMO guidelines [51], neoadjuvant treatment for downstaging is indicated in case of primary non-resectability, with evaluation of tumor regression in 3–4 monthly intervals. Surgical resection is then scheduled once maximal radiological tumor regression is achieved. Based on data of 3 RCTs, adjuvant therapy for 3 years is recommended for patients with high-risk tumors [50], [52], [53], [54]. In contrast, tyrosine kinase-inhibitors should be continued indefinitely in locally advanced or ruptured GIST due to high-rates of recurrence until disease progression occurs [55], 56], similar as in the palliative setting for metastatic GIST. In cases of irresectability, imatinib has the potential to significantly prolong median survival [57].

### Granular cell tumor

Granular cell tumors (GCT) are yellowish polypoid lesions that originate from the submucosa and may show malignant transformation in 1–2 % of cases [58]. Microscopically, GCTs consist of nests of polygonal cells resembling histiocytes with PAS-positive granules separated by collagen bundles. S-100 immunohistochemistry is usually positive. Malignant cases have a poor prognosis and show necrosis, increased mitotic count, large hyperchromatic nucleoli, and a high Ki67 index [59], [60], [61], [62]. Lesions <1 cm can often be surveilled, whereas tumors up to 3 cm may be resected endoscopically, with surgery reserved for larger tumors [58], 63].

### Schwannoma

Schwannomas are rare, usually benign, mesenchymal lesions originating from the Schwann cells of the nerve sheath which are found in all human soft tissues [63], [64], [65]. They occur in the 4th – 5th decade of life with a slight predominance in females. Like GCT, Schwannoma originate from the submucosal layer [66] with a yellowish, smooth, firm endoscopic appearance, and show strong expression of S-100 and SOSX10 on immunohistochemistry. Tumors >2 cm should be resected, while malignant transformation is exceedingly rare [67].

### Hemangioma

Esophageal hemangioma is a rare and mostly solitary benign lesion with an overall prevalence of 0.04 % in autopsy series [1], 66]. Endoscopically, they appear as blue lesion, while biopsy should not be undertaken due to bleeding risk. Multiple hemangioma are typical for blue rubber bleb nevus (Bean’s) syndrome, a congenital disease associated with multiple skin and gastrointestinal lesions [68]. Most esophageal hemangioma are asymptomatic and found incidentally [69], but intestinal hemorrhage and dysphagia may occur and call for individualized treatment, ranging from iron supplementation and blood transfusions to endoscopic or surgical resection [69], 70].

### Lipoma/fibrovascular polyps

Lipomas account for less than 1 % of mesenchymal esophageal tumors. More than 85 % occurs in the proximal third of the esophagus, where they typically present as fibrovascular polyps with a pedunculated intraluminal growth pattern [34], 71], 72]. In contrast, the less common intramural esophageal lipomas are usually small, asymptomatic, and typically located in the lower third of the esophagus. Lipomas are usually discovered incidentally on radiologic imaging or endoscopy, but pedunculated proximal polyps/lipoma carries a risk of asphyxia [73]. Microscopically, esophageal lipomas have mature adipose tissue with fibrovascular septa; given the potential for malignant transformation, MDM2 *in situ* hybridization is recommended [74], 75]. In a recent systematic review, 63 of 176 cases of esophageal lipomas were ultimately diagnosed as liposarcoma [34].

### Glomus tumor

Glomus tumors originate in the glomus apparatus, an arterio-venous shunt of the dermis involved in physiologic thermoregulation of the body. Esophageal glomus tumors are extremely rare, with fewer than 20 cases described in the literature [76], 77]. They arise in the deep mucosa and submucosa and show epithelioid cells with associated proliferation of small blood vessels on histopathological examination. Of note, malignant transformation including distant metastasis has been described [77].

## Therapeutic management of SET

Asymptomatic SET without high-risk stigmata discovered incidentally usually do not require specific treatment. However, depending on the size of the lesion and extent of extension into the esophageal lumen, dysphagia, bleeding/ulceration, and thoracic pain may occur. The indication for resection may also be due to compression of extraesophageal organs by large tumors or the risk of asphyxia in pedunculated polyps in the upper esophagus [34]. The decision to resect should be made in a multidisciplinary meeting. The choice of technique should depend on size, location, and local expertise [12]. Treatment options include interventional-endoscopic and surgical procedures up to esophagectomy [6].

### Surveillance

According to the current ESGE guidelines, endoscopic surveillance of asymptomatic esophageal leiomyomas, lipomas, granular cell tumors, schwannomas, and glomus tumors is generally not required [12]. In contrast, asymptomatic SET without a clear diagnosis should be followed up endoscopically. In this regard, the recommended frequency of surveillance endoscopies is based on size: 2–3 years for lesions <10 mm and 1–2 years for lesions of 10–20 mm. For asymptomatic SEL >20 mm that are not resected, ESGE recommends surveillance with EGD plus EUS at 6 months and then at 6–12-month intervals [12].

### Endoscopic therapy

Endoscopic resections have become a standard procedure to remove superficial/epithelial SET (papillomas, adenomas, inflammatory polyps) as well as deeper submucosal and even transmural tumors. Mucosal lesions are elevated by saline injection, resulting in detachment of deeper tissue layers, and then resected by snare removal, ligation and electrocautery; larger lesions may be removed by piece-by-piece technique [78]. Potential complications of endoscopic mucosal resections include bleeding, perforation and stenosis in up to 88 % [79].

Endoscopic submucosal resections follow a similar principle with opening of the submucosal space and are generally more difficult and associated with higher complication rates than mucosal resections. Relevant bleeding occurs in up to 5 %, especially in lesions >4 cm and patients on anticoagulation, while perforations are reported in up to 10 %, but can directly be treated via application of clips [80].

Another endoscopic modality is peroral endoscopic tumor resection (POET), which is derived from POEM. After incision of the mucosa approximately 5 cm proximal to the lesion, the submucosal space is tunneled for tumor removal. Indications for POET are usually tumors up to 3 cm [81].

### Surgical therapy

In the past, open transthoracic enucleation of the SET was associated with significant morbidity. However, with the advent of minimally invasive and robotic procedures, postoperative morbidity and mortality have decreased significantly [36], [82], [83], [84], [85]. Larger tumors >5 cm or lesions encircling the esophagus have been cited as limiting factors of thoracoscopy or laparoscopy [35], but minimally invasive surgery has also been described for tumors of much larger dimensions [84].

Tumor enucleation is an effective treatment for most SET and is technically feasible in up to 97 % [82]. It is the standard surgical approach for esophageal leiomyoma [83]. Because these lesions have a benign clinical course with a very low rate of malignant transformation, recurrence rates after surgery are negligible when appropriate surgical techniques are used [35], 82], 84]. In contrast, recurrence rates can be higher in GIST, especially in larger tumors with high mitotic activity [86], 87]. Therefore, the optimal therapy for GIST remains debated, and current recommendations range from enucleation to partial or subtotal esophagectomy [25], 88], 89]. Nevertheless, in cases with low mitotic index and smaller size (<4 cm), retrospective series indicate that enucleation is a viable treatment option for GIST [87], [90], [91], [92].

**Figure 2:**
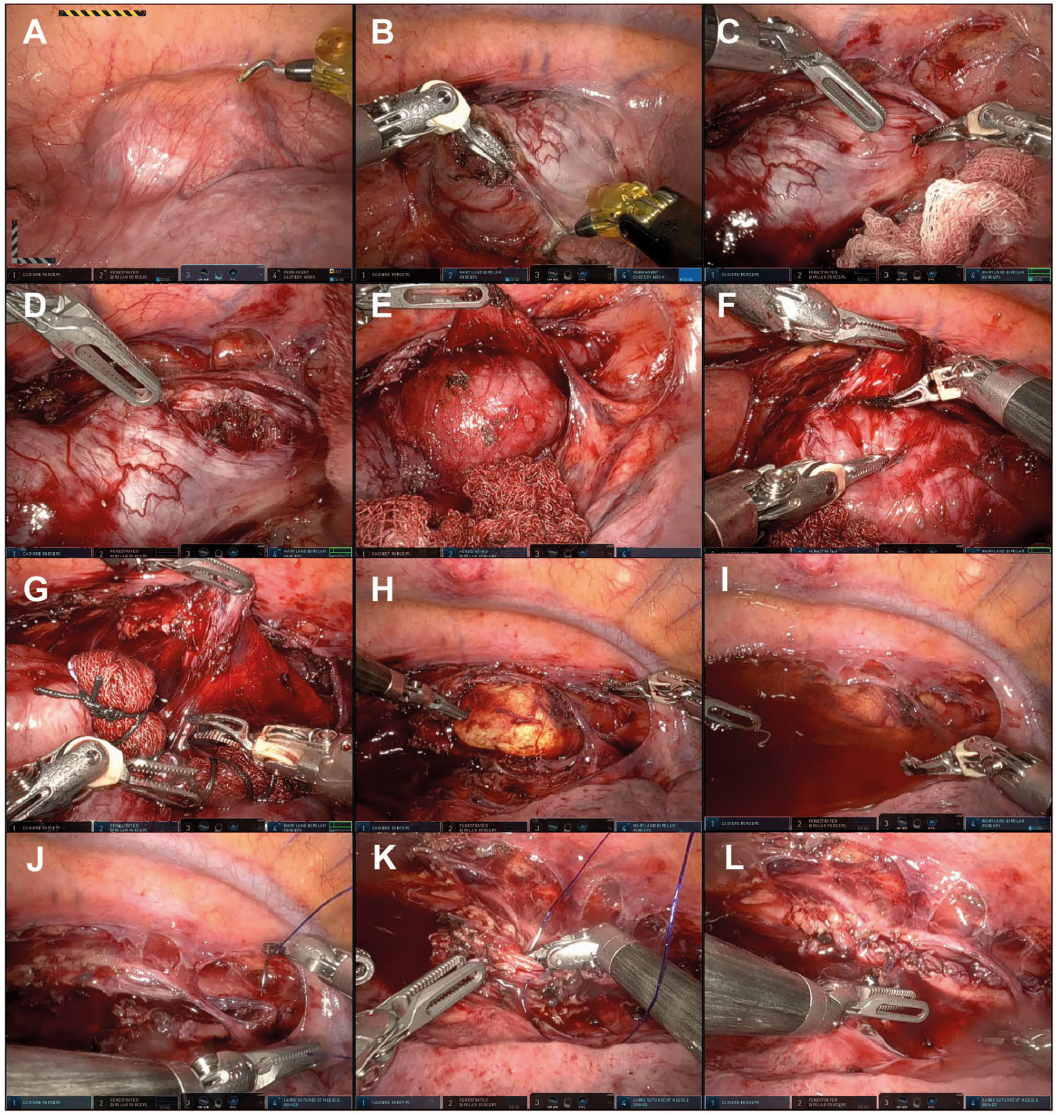
Steps of a robotic assisted minimally invasive enucleation of the esophageal GIST of the patient presented in Figure 1. (A) initial view, (B) incision of pleura/serosa; (C, D) length incision of esophageal muscle layer, (E, F) circular detachment of the tumor, (G) complete en-bloc removal, (H) checking mucosal integrity by endoscopy, (I) checking mucosal integrity by underwater air leakage test during endoscopy, (J, K) closure of muscle defect by monofilament barbed suture, (L) final view.

From a surgical-technical point of view, intraoperative endoscopy is valuable for exact determination of tumor localization and control of mucosal integrity [93]. In addition, most authors advocate closure of the muscle layer defect after enucleation to prevent the development of pseudodiverticula [83], 94]. Figure 2 shows the main steps of a robot-assisted thoracic enucleation of a distal esophageal GIST.

**Figure 3:**
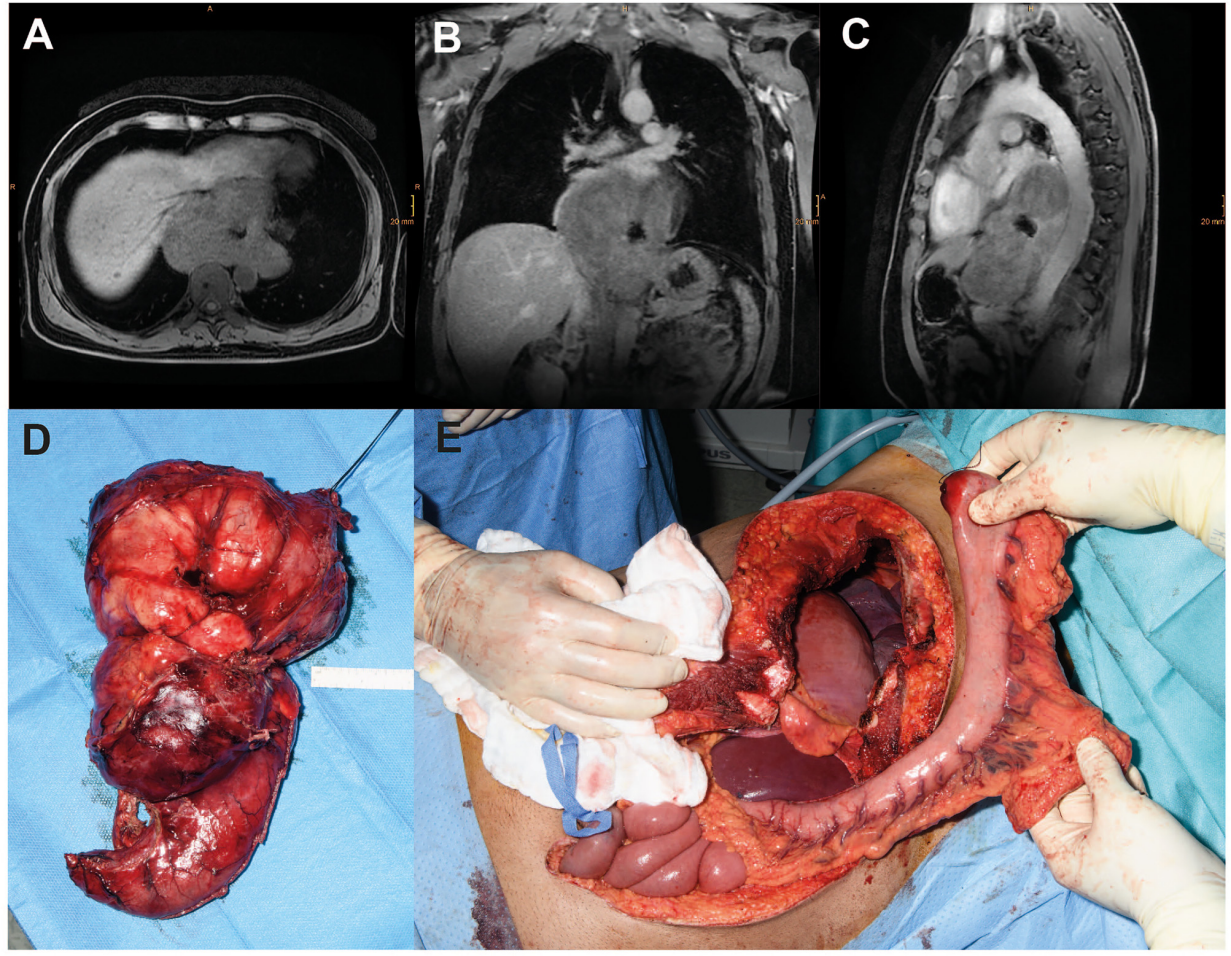
Case of a male patient with a giant (17 × 14 × 8 cm) esophageal leiomyoma with abscess following diagnostic biopsy. Transverse (A), coronal (B) and sagittal (C) magnetic resonance imaging planes of the tumor. (D) Intraoperative images of the resected specimen and (E) the situs after thoraco-laparotomy before reconstruction with interposition of the tubulized stomach.

SET above a certain size, specific entities (e.g., malignant GIST with mucosal infiltration), or complications (e.g., central necrosis or abscess formation after biopsy) may not be treatable by enucleation. For oncologic reasons, esophagectomy may also be required for GIST with high-risk stigmata [45]. Figure 3 shows the case of a patient with a large esophageal leiomyoma. Due to the size of the lesion and involvement of the esophagogastric junction with large tumor nodules on both sides of the diaphragm, open surgical thoraco-laparotomy was required for en bloc tumor removal, and continuity was reconstructed with a gastric pull-up procedure and anastomosis at the level of the azygos vein according to Ivor Lewis.

## Conclusions

SET is an umbrella term for various rare sub-entities, and solid knowledge of the histopathological spectrum, the diagnostic work-up and entity-specific treatment options is necessary for an adequate clinical decision-making. A multidisciplinary tumor board should be generally involved in the therapeutic management of SET.

Small asymptomatic SET usually requires only endoscopic surveillance, while therapy is typically indicated based on symptoms, tumor size or growth, or unclear diagnosis. Most SET are benign with excellent long-term outcome, but malignancy may be present in gastrointestinal stroma tumor (GIST), and rarely even in Schwannoma, granular cell tumor, or leiomyoma. For leiomyoma, surgical resection is generally only indicated in symptomatic cases, while intermediate to high-risk GIST should undergo resection. Therapeutic options range from endoscopic interventions to major surgical procedures such as esophagectomy and multimodal strategies. However, enucleation via a minimally invasive or robotic-assisted approach remains the standard of care for most SET sub-entities.
